# Gibberellic Acid Signaling Is Required to Induce Flowering of Chrysanthemums Grown under Both Short and Long Days

**DOI:** 10.3390/ijms18061259

**Published:** 2017-06-12

**Authors:** Bin Dong, Ye Deng, Haibin Wang, Ri Gao, Githeng’u K. Stephen, Sumei Chen, Jiafu Jiang, Fadi Chen

**Affiliations:** College of Horticulture, Nanjing Agricultural University, Key Laboratory of Landscape Agriculture, Ministry of Agriculture, Nanjing 210095, China; maddonbin@gmail.com (B.D.); 2013104105@njau.edu.cn (Y.D.); hb@njau.edu.cn (H.W.); 2013204031@njau.edu.cn (R.G.); 2015204038@njau.edu.cn (G.K.S.); chensm@njau.edu.cn (S.C.)

**Keywords:** gibberellin, photoperiod, mutant, floral induction, flowering time

## Abstract

Flower bud formation and flowering in chrysanthemums occur under short day conditions (SD), but the molecular basis for the switch to reproductive growth is less well understood than in model plants. Here, a spontaneous mutant able to flower under long days is described. In an attempt to reveal the pathway(s) involved in the formation of flower buds under contrasting daylengths, transcriptome sequencing was carried out in plants grown both under SD and long day conditions (LD). A number of differentially transcribed genes involved in the various known flowering pathways were identified. Both circadian clock genes and *Chrysanthemum FLOWERING LOCUS T Like3* (*CmFTL3*) were up-regulated under SD, thereby inducing floral bud formation and flowering. The gibberellin (GA) signaling pathway-related genes *Gibberellin 20-oxidase* (*GA20ox*) and *Gibberellin receptor* (*GID1*) were up-regulated in the mutant under LD, while the catabolic genes *Gibberellin 2-oxidase* (*GA2ox*) and *GA-INSENSITIVE* (*GAI*) were down-regulated, thereby inducing the transcription of *SUPPRESSOR OF OVEREXPRESSION OF CONSTANS 1* (*SOC1*) and *LEAFY* (*LFY*). The GA content of the leaf was higher in the mutant than in the wild type (WT) under LD and SD, and the mutant has more branching than WT plants under LD or SD. When treated with GA, the mutant flowered earlier under both SD and LD relative to WT, but there was no detectable phenotype difference between the two lines. The indication was that the photoperiod pathway majorly regulates flower bud formation and flowering time in chrysanthemums under SD. The GA signaling pathway only plays a subsidiary role for flowering. However, the GA signaling pathway predominated for flowering under LD.

## 1. Introduction

Flowering is a key event in both seed and ornamental crops [1]. The switch to reproductive growth occurs in response to a variety of endogenous and environmental cues, and in the model plant *Arabidopsis thaliana* at least, six major pathways (photoperiod, vernalization, autonomous, gibberellin (GA), ambient temperature and aging) are known to regulate the floral transition process [2,3]. In nature (except at the Equator), the photoperiod varies throughout the year, and plants sense its length through certain photoreceptor molecules [4,5]. The genes of circadian rhythms (including *GIGANTEA*, *GI*; *TIMING OF CAB transcription 1/PSEUDO-RESPONSE REGULATOR 1*, *TOC1/PRR1*; *CIRCADIAN CLOCK ASSOCIATED 1*, *CCA1* and *LATE ELONGATED HYPOCOTYL*, *LHY*) regulate CONSTANS (CO) and thus mediate flowering [6,7]. FT is transcribed in the leaf vasculature under long day conditions (LD); when its transcript reaches the shoot apical meristem, both *APETALA1* (*AP1*) and *LFY* are induced, which in turn triggers flowering [8,9].

Gibberellins (GAs) are biosynthesized from *ent*-kaurenoic acid via *ent*-kaurene; *ent*-copalyl diphosphate synthase (*CPS*) plays an important role in GA formation [10]. GA makes an important contribution to the switch to reproductive growth [11]. The activity of *GA20ox* determines the endogenous concentration of GA in the stem apical meristem, while *GA2ox* catalyzes GA inactivation [12,13]. GA regulates *SOC1* and *LFY* gene activity; overexpression of *SOC1* rescued GA-deficient mutant *ga1-3* to induce flowering [14]. GA promoted *FT* expression independent of *CO* genes under LD [15]. GA suppresses the production of the DELLA protein (including *GAI*; *REPRESSOR OF GA1-3*, *RGA1-3*; *RGA-LIKE1-3*, *RGL1-3*), which is antagonistic to GA signaling [16,17]. In addition, members of other transcription factor families, namely MYB, MADS, AP2/ERF, bHLH, WRKY and TCP, are also involved in floral induction and flowering [18,19,20,21,22]. For example, *FLOWERING LOCUS C* (*FLC*) transcription is regulated by the MADS box transcription factor *SHORT VEGETATIVE PHASE* (*SVP*), the product of which binds to the promoters of *FT* and *SOC1* [3,23].

Chrysanthemum (*Chrysanthemum morifolium*), a highly prized ornamental species, is a short day plant [24], which typically flowers in the fall. Its genome harbors three *FT* orthologs, termed *FTL1* through *3. FTL3* appears to encode a major component of the photoperiod pathway, which promotes flowering under SD [25]. Transcription of the *FTL*s is repressed under LD. Recently, a spontaneous mutant of the chrysanthemum variety ‘Jinba’ was discovered that was able to flower under LD. Here, the effect of GA on the flowering behavior of the mutant was examined, and a transcriptomic analysis was carried out to identify the genes, which are differentially transcribed between the mutant and the wild type.

## 2. Results

### 2.1. Flowering Behavior of the ‘Jinba’ WT and Mutant in Response to GA Treatment

**Figure 1:**
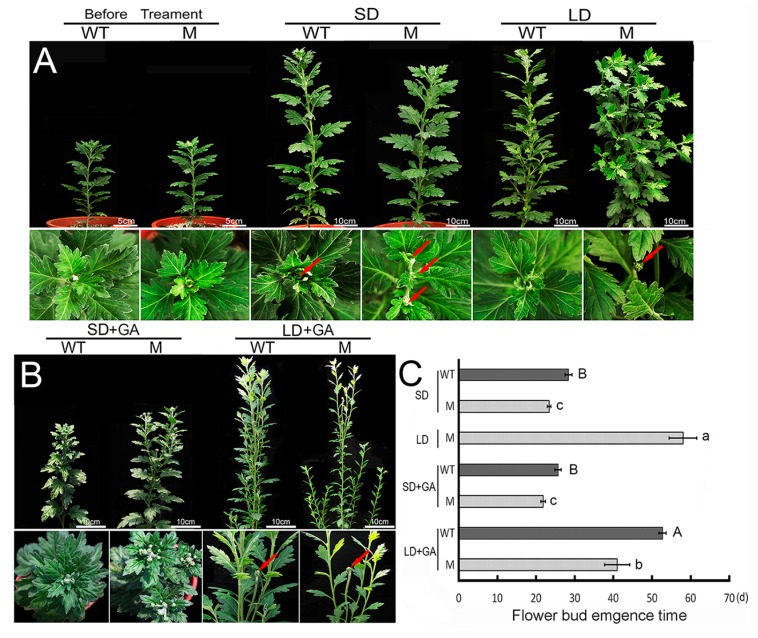
The switch to reproductive growth in WT and M chrysanthemum plant. (**A**) Flower bud emergence (arrowed) in plants grown under LD or SD; (**B**) Flower bud emergence (arrowed) induced by spraying plants with GA under LD or SD; (**C**) The effect of GA application on the timing of flower bud emergence. Significant differences were determined by Duncan’s multiple range test (*p* < 0.05); error bar indicates SD. Different uppercase (**A**,**B**) or lowercase (a–c) letters indicate significant differences in the time required for WT and M plants, respectively.

**Figure 2:**
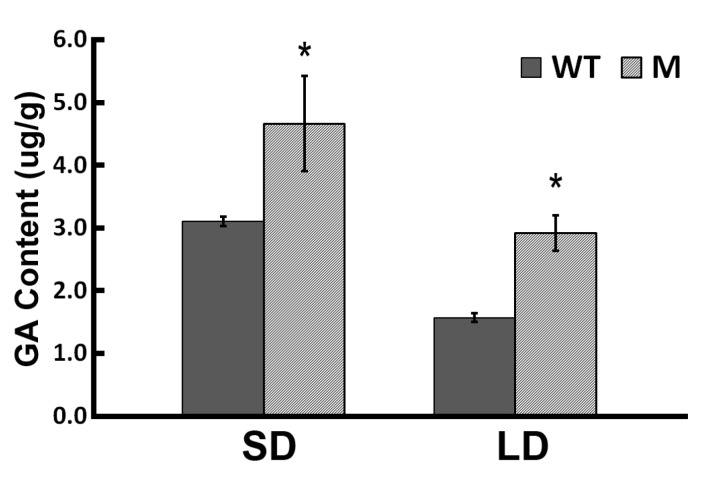
GA content in the leaves of WT and M plants grown under LD or SD. Significant differences were determined by Duncan’s multiple range test (*p* < 0.05, *n* = 3). * means significantly difference.

The timing of flower bud emergence of both the WT and mutant (M) forms of ‘Jinba’ was investigated by growing them under both SD and LD (Figure 1A). Under SD, the first flower buds emerged in 29 and 24 days, respectively (Figure 1C). Under LD, reaching this stage took about 58 days, while WT plants never flowered (data not shown). The endogenous concentration of GA was determined in both young leaves of WT and Mutant (M) plants grown under either LD or SD (respectively, WT-SD, M-SD, WT-LD and M-LD) and those of WT and M plants still in the vegetative stage, sampled prior to the imposition of a controlled photoperiod (Figure 2). Under LD, the GA concentration was substantially higher in M than in WT plants, but there was no significant difference between M and WT in plants grown under SD (Figure 2). When GA was sprayed on the leaf surface of the plants (Figure 1B), the time taken to reach flower bud emergence was reduced to 26 days for WT and to 22 days for M in plants grown under SD, while under LD it was 53 and 41 days, respectively (Figure 1C).

### 2.2. Transcriptome Sequencing and Bioinformatic Analysis

The transcriptome of both WT and M plants grown under either LD or SD was sampled when the M plants were at flower bud differentiation stage, but the WT plants remained either vegetative (LD) or were flowering (SD). Four cDNA libraries were assembled (M-LD, WT-LD, M-SD and WT-SD) and sequenced using the RNA-Seq platform. A total of 20,897,890,920 nt was acquired (Table 1), from which 107,596 unigenes were identified. The unigenes together represented 110,025,243 nt of sequence, and were of mean length 1023 nt; the N50 was 1600 nt. About 60% of the unigenes have been previously annotated: 61,666 in the NR database, 45,863 in NT, 42,262 in Swiss-Prot, 39,147 in KEGG, 23,818 in COG and 46,064 in GO. The total number of coding sequences identified was 65,257 including 60,752 mapped to the protein database and 4505 coding sequences are predicted. An analysis of those represented in the NR database showed that 62% were associated with other sequences at an *E*-value less than 1 × 10^−30^, while 73% of them shared between 80–100% identities (Figure S1A,B). The most commonly occurring species harboring these matching sequences was grapevine (26.0%), followed by tomato (16.6%) (Figure S1C). A COG analysis divided the unigenes into 25 categories (Figure S2A); the largest group was “general functions” (group R, 17.7% of the genes), followed by “replication, recombination and repair” (group L, 9.4%) and “transcription” (group K, 9.2%). A GO analysis identified the unigenes as being involved in 22 biological processes, 17 cellular components and 16 molecular functions (Figure S2B).

### 2.3. The Transcription Behavior of Genes Involved in the Determination of Flowering Time

**Figure 3:**
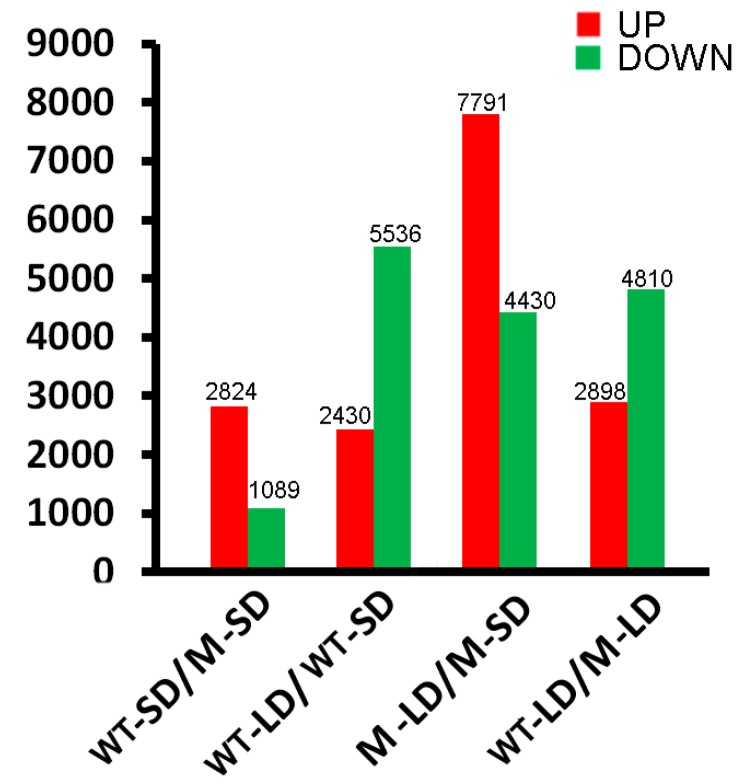
Numbers of differentially transcribed genes in WT and M plants grown under LD or SD. WT-SD represents wild type plants under short day conditions, WT-LD represents wild type plants under long day conditions, M-SD represents mutant plants under short day conditions, and M-LD represents mutant plants under long day conditions.

A representation of the genes which were differentially transcribed in the contrasts WT-LD/WT-SD, M-LD/M-SD, WT-SD/M-SD and WT-LD/M-LD is given in Figure S3. Differential transcription was defined as the ratio of transcript abundance being either >2 (up-regulation) or <0.5 (down-regulation), along with a *p* value of <0.001. In all, in the contrast WT-SD/M-SD, 2824 transcripts proved to be more abundant and 1089 less abundant in WT, while in WT-LD/M-LD, the equivalent numbers of transcripts were 2898 and 4810, respectively. In the contrast WT-LD/WT-SD, 2430 genes were up-regulated and 5536 down-regulated, while in M-LD/M-SD, the equivalent numbers of genes were 7791 and 4430, respectively (Figure 3, Figures S3 and S4).

A particular focus was placed on genes involved in the photoperiod, GA signaling, aging, ambient temperature and autonomous pathway (Table 2 and Table S1). The M-LD/WT-LD was of interest since plants in the latter category reached flowering, while those in the former category did not. The photoperiod pathway genes (*TOC1*, *PRR5*, *LHY*, *COL* and *CmFTL3*) were not up-regulated in M, but three genes belonging to the GA pathway (*GA20ox*, *GA2ox* and *GID1*) were. In addition, *CmFTL1* transcript abundance was lower in both WT and M plants grown under SD than in the same genotypes grown under LD. Both *SOC1* and *FLO/LFY-like* transcripts were noticeably more abundant in M-LD than in WT-LD, suggesting their involvement in the capacity of M plants to flower under LD. In M-SD/WT-SD, *TOC1*, *LHY*, *PRR5*, *COL*, *SOC1* and *CmFTL3* were all more strongly transcribed in M than in WT. The GA pathway gene *CPS* strongly increased in M-SD/WT-SD, while the other genes involving *GA20ox*, *GA2ox* and *GID1* showed no significant differences in M-SD/WT-SD. In addition, the gene *SQUAMOSA PROMOTER BINDING PROTEIN-LIKE5* (*SPL5*) was similarly up-regulated in both plant types under LD. Transcription of the inhibition flowering gene *SVP* was strongly suppressed in both WT and M plants under SD. The transcript abundance of *FLC* and *FRI* was higher in M plants grown under SD than in those grown under LD.

### 2.4. Differentially Transcribed Transcription Factors

The differential transcription of members of the transcription factor families MYB, MADS, AP2/ERF, bHLH, WRKY and TCP (Table S2) was considered to be significant, as their products are known to make an important contribution to plant development. To be designated as differentially transcribed, the threshold ratio of transcript abundance was required to be >2 (up-regulation) or <0.5 (down-regulation), and the *p* value < 0.001. In WT-SD/WT-LD, 24 MYB, 10 MADS-box, 1 AP2/ERF, 13 bHLH, 12 WRKY and 1 TCP transcription factors were differentially transcribed, while in the same contrast in M (M-SD/M-LD), there were 39 MYB, 7 MADS-box, 1 AP2/ERF, 13 bHLH, 29 WRKY and 6 TCP transcription factors. The comparison M-SD/WT-SD revealed the up-regulation of 5 MYB, 5 MADS-box, 4 bHLH and 1 TCP transcription factor, while in M-LD/WT-LD, 9 MYB, 4 MADS-box, 3 bHLH and 19 WRKY transcription factors were up-regulated.

### 2.5. qRT-PCR-Based Validation of RNA-Seq Acquired Transcript Abundance

**Figure 4:**
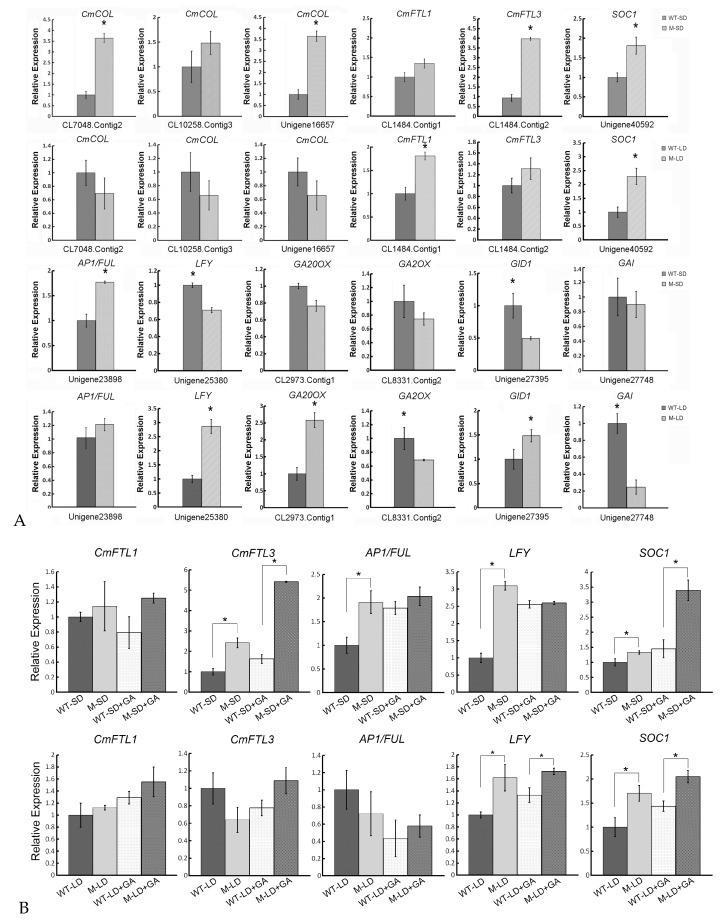
Transcription of key flowering-related genes using qRT-PCR. (**A**) Relative transcript abundance in WT and M plants grown under LD or SD; (**B**) Relative transcript abundance in WT and M plants sprayed with GA and grown under LD or SD. * indicates significantly different expression level between two samples. Student’s *t* test (*n* = 3).

Validation of the differential transcription of the photoperiod and GA pathway genes derived from the RNA-Seq analysis was obtained using qRT-PCR (Figure 4). The analysis showed that *CmCOL* (CL7048.Contig2, Unigene16657), *CmFTL3* (CL1484.Contig2), *SOC1* (Unigene40592), *AP1/FUL* (Unigene23898) and *LFY* (Unigene25380) were all more strongly transcribed in M-SD than in WT-SD. However, the GA pathway genes involving *GA20ox* (CL2973.Contig1), *GA2ox* (CL8831.Contig2) and *GAI* (Unigene27748) showed no significant difference; only *GID1* (Unigene27395) appeared to be differentially transcribed in M-SD. Under LD conditions, the abundance of *CmFTL1* (CL1484.Contig1), *GA20ox* (CL2973.Contig1), *GID1* (Unigene27395), *SOC1* (Unigene40592) and *LFY* (Unigene25380) transcript was markedly greater in M-LD than in WT-LD. The expression of catabolic gene *GA2ox* (CL8831.Contig2) and *GAI* (Unigene27748) showed down-regulation in mutant plants under long days, while there was no evidence for the differential transcription of *CmCOL* (CL7048.Contig2, CL10258.Contig3, Unigene16657) and *CmFTL3* (CL1484.Contig2).

Following GA treatment, compared with the WT, *SOC1* and *LFY* were both more strongly induced in mutant plants either in short days or in long days, while *CmFTL3* showed significant induction in short days (Figure 4B). The gene *AP1/FUL* showed significant up-regulation in short days, but no significant difference in long days.

## 3. Discussion

### 3.1. The Photoperiod and GA Pathways Act in Concert to Regulate Flowering Time under SD

Photoperiod is one of the most important environmental cues determining flowering time. The key circadian clock protein genes are *TOC1*, *CCA1*/*LHY* and the *PRRs* [2,7]; the present data have revealed that the range of circadian clock genes (*CmTOC1*, *PRR5*, and *LHY*) and *CmCOL* were all strongly up-regulated in M-SD/M-LD and WT-SD/WT-LD. For the chrysanthemum *FTL* orthologs, when *CmFTL3* is over-expressed, the plant flowers early even under non-inductive conditions [25]. While both *CmFTL3* and *SOC1* transcription was enhanced by SD in WT relative to WT-LD, in M only *CmFTL3* behaved in this way. The inference is that the repressor of flowering of the up-regulated *FRI* and *FLC-like* genes might affect the transcription of *SOC1* in M-SD (Table 2). In addition, *CmFTL3* transcription was significantly higher in M-SD than in WT-SD, resulting in the mutant reaching flowering earlier than the WT, consistent with the suggestion of Oda [25]. *CmFTL1* was only weakly transcribed in both WT and M plants under SD, while the abundance of *AP1/FUL* was enhanced by SD in WT and even more so in M, and *FLO/LFY*-*like* was up-regulated by LD in both genotypes. Overall, the photoperiod pathway appeared to have a major regulatory influence over the switch to reproductive growth in the short day conditions.

The GA signaling pathway was also a significant determinant of flowering time in both WT and M. The endogenous level of GA responded positively to SD in both WT and M, although it was markedly higher in the latter genotype (Figure 2). Compared with LD, the GA synthesis gene *GA20ox* was up-regulated in both WT and M under SD; the product of this gene is known to both support GA synthesis in the leaf and enhance the plant’s response to GA signaling [26]. Besides promoting vegetative growth, GA activates *FTL* to promote early flowering in *A. thaliana*. In the *cpd ga20ox* double mutant of *A. thaliana*, the content of GA is greatly reduced, and flowering is delayed [12,27]. It has also been established from the behavior of *A. thaliana* plants grown under SD that GA induces *SOC1* transcription to promote flowering [14,28]. In chrysanthemums, the loss of BBX24 (a zinc finger transcription factor) not only accelerates flowering, but also induces the photoperiod and GA synthesis pathways, which activates *CmFLT3* but not *CmFTL1* transcripts [29]. Meanwhile, the transcription of the catabolic genes *GA2ox* and *GAI* is inhibited in WT and M plants under SD. The implication is that the GA signaling pathway might affect flowering under SD via its effect on *CmFTL3* and/or *SOC1*. These models derive from *Arabidopsis* and they are not fully demonstrated in chrysanthemums.

### 3.2. GA Activates SOC1 to Promote Flowering under LD

The chrysanthemum is considered a short day plant; it generally blooms in the fall, since floral induction only begins once the daylength begins to shorten. The mutant, however, is able to flower under LD, implying a change to a gene product that is a component of the daylength-induced floral induction mechanism. The transcriptomic data showed that both *GA20ox* (GA synthesis) and *GID1* (GA sensing) were more strongly transcribed in M than in WT under LD, while the GA catabolism genes *GA2ox* and *GAI* were relatively less strongly transcribed (Table 2, Figure 4A). At the same time, *SOC1* and *LFY* were up-regulated in M, but the genes involved in the sensing photoperiod were not differentially transcribed. Endogenous GA levels were higher in M than in WT (Figure 2), while the exogenous supply of GA induced flowering in both M and WT under LD, with M plants reaching flowering before the WT plants did (Figure 1B,C). The implication is that the GA pathway regulates floral induction under LD, operating via *SOC1* and *LFY*. A previous report showed that the transcript level of *CmFTL3* was up-regulated specifically by GA [29], which might be due to the different genetic background of cultivars.

### 3.3. Transcription Factors Involved in Floral Induction in Chrysanthemum

Plants have evolved a large number of transcription factors in order to regulate growth, development and differentiation [30]. Here, five major transcription factor families were targeted in the transcriptomic analysis, namely MYB, MADS, AP2/ERF, bHLH, WRKY and TCP (Table S2). Four *MYB2-like* genes have been described (all homologous to *AtMYB44*) that negatively regulate flowering in chrysanthemum [31]. Here, the comparison between the WT and M transcriptomes identified a *MYB2* transcript that was more strongly transcribed under LD in WT than in M, whereas under SD its abundance was comparable in the two genotypes. The suggestion is therefore that this transcription factor is involved in delaying flowering time under LD. Among the MADS transcription factors, both an *AP1/FUL* homolog (Unigene23898) and *CDM41* (Unigene7813) were significantly up-regulated in the WT by SD, and thus may be involved in floral induction under SD. In M plants, the same *AP1/FUL* homolog, along with the *AP1* homolog Unigene55133, was more strongly transcribed under SD than under LD. *AP1/FUL* is a key regulator of floral development in plants [32], while *CDM41* belongs to the *FUL* clade of MADS genes [33]. The *AP2/ERF* factors are known to participate in floral development in many plant species, as well as in the stress response [34,35]. An *AP2/ERF* homolog (Unigene23878) was found in both WT and M to be more abundantly transcribed under SD than under LD, so it may well be part of the floral induction mechanism under SD. Two *AP2/ERF* homologs (Unigene47162, Unigene46823) were also more strongly transcribed in M than in WT under LD, so the possibility is that the activity of *AP2/ERFs* underlies the ability of M plants to flower under LD. No representatives of the bHLH, WRKY or TCP families were differentially transcribed in any of the contrasts analyzed.

## 4. Materials and Methods

### 4.1. Plant Materials and Growth Conditions

The M forms of ‘Jinba’ were found during cut flower production, which showed earlier flowing than the WT plants under SD, and an abnormally emerged flower bud in LD. Both are maintained by the Nanjing Agricultural University Chrysanthemum Germplasm Resource Preserving Centre (Nanjing, China). Uniform chrysanthemum cuttings were raised in a greenhouse under natural light at ~25 °C, and a relative humidity of 70–75%. After 20 days (when the plants had reached a height of ~20 cm and were still vegetative), the fifth fully expanded leaf of each plant was sampled. Two contrasting photoperiod regimes were then applied: LD (~16 h light/8 h dark) and SD (~8 h light/16 h dark). The fifth fully expanded leaves were sampled randomly from six lines of M-SD/LD and M-SD/LD at the same time stage when the M plants had visible flower buds. The flower bud differentiation, stem apices of M and WT plants were observed by using paraffin sections. Immediately after harvest, the tissue was snap-frozen in liquid nitrogen and stored at −80 °C until required. Three biological replicates for the experiment were performed.

### 4.2. RNA Extraction and cDNA Library Construction

Total RNA was isolated from 0.3 g of tissue samples using the RNAiso Plus reagent (TaKaRa, Dalian, China), following the manufacturer’s protocol. The resulting crude RNAs were treated with RNase-free DNase I (TaKaRa) and their concentrations measured using an HND-1000 NanoDrop device (http://www.nanodrop.com/). The OD_260/280_ of the RNAs lay between 1.8 and 2.2 and the OD_260/230_ was >1.8. According to the method of Singh et al. [36], a 3 μg pool of RNA was formed by combining 1 μg from each biological replicate used for the construction of libraries. The four RNA-Seq libraries were constructed as described by Ren et al. [37].

### 4.3. Transcriptome Sequencing and Bioinformatic Analysis

Transcriptome sequencing was carried out using a Hiseq 2000 device (Illumina, San Diego, CA, USA) at the Beijing Genomics Institute (www.genomics.cn). Image data output from a sequencing machine was transformed by base calling into sequence data, which is called raw reads and stored in fastq format. Raw reads contain “dirty” reads (adapters, unknown or low quality bases). The clean reads were edited by removing adaptor sequences (match length ≥ 10 bp); unknown bases are more than 5% and low quality reads (the percentage of low quality bases (the value of sQ ≤ 20) is over 50% in a read; we define the low quality base whose sequencing quality is no more than 10). Then transcriptome de novo assembly was carried out with a short-reads assembling program—Trinity (release-20130225, http://trinityrnaseq.sourceforge.net/). The produced sequences of trinity are called unigenes. The unigenes using BLAST software (v2.2.26 + x64-linux, http://blast.ncbi.nlm.nih.gov/Blast.cgi) were assigned functions based on homologs present in the NR (www.ncbi.nlm.nih.gov/refseq), NT (www.ncbi.nlm.nih.gov/nuccore), Swiss-Prot (www.uniprot.org), KEGG (www.genome.jp/kegg/kegg1.html), COG (www.ncbi.nlm.nih.gov/COG/) and GO (geneontology.org/page/go-database) databases. For the prediction of coding regions, the number of CDS that mapped to the protein database and the number of CDS were predicted. In addition, transcript abundance was estimated using the RPKM reads (clean reads per kilo base per million) method on the base of eliminating the influence of different gene length and sequencing discrepancy using RPKM (internal software of BGI) and SOAP (Release 2.21, http://soap.genomics.org.cn/soapaligner.html). The RPKM values can be directly used for comparing the difference of gene expression among samples. Differential transcription was defined as the ratio of transcript abundance being either >2.0 (up-regulation) or <0.5 (down-regulation), along with a *p* value of <0.001 [38]. The datasets of transcriptome (Chrysanthemum. ‘Jinba’ sequencing project) are available in the NCBI repository, (http://trace.ncbi.nlm.nih.gov/Traces/sra_sub/sub.cgi?) Accession No. for library SRP070731.

### 4.4. qRT-PCR

Total RNA was extracted from the leaves and the shoot apex using the RNAiso Plus reagent (TaKaRa), and the first cDNA strand synthesized from 1 μg total RNA using PrimeScript^®^ Reverse Transcriptase (TaKaRa) primed with oligo (dT), following the manufacturer’s protocol. Each 20 μL qRT-PCR contained 10 μL SYBR^®^ Premix Ex Taq^TM^Ⅱ, 5 μL cDNA and 1 μL of forward primer and 1 uL of reverse primer, 3 μL H_2_O. The primers were designed using primer 5.0 software (sequences are given in Table S3). Each reaction was initially denatured (95°C/2 min), then subjected to 40 cycles of 95 °C/15 s, 55 °C/15 s, 68 °C/20 s and performed in triplicate. The chrysanthemum *EF1α* gene (Genbank accession number KF305681) was used as the reference [39]. Relative transcript abundances were calculated using the 2^−∆∆*C*t^ method [40]. Three biological replicates were performed.

### 4.5. Determination of Endogenous GA Content

Three replicates of 1 g leaf samples (third or fourth fully expanded leaf) were obtained under both LD and SD conditions. At the time of sampling, WT-LD was at the vegetative stage, while WT-SD, M-SD and M-LD were at the flower bud differentiation stage. The leaf material was powdered by grinding it in the presence of liquid nitrogen. The samples were extracted by suspension in 5 mL 80% (*v/v*) cold methanol and centrifuged (10,000 rpm/20 min, 4 °C); 0.2 g PVP was then added and the suspension left for >1 h at 4 °C. After re-centrifuging (10,000 rpm/20 min, 4 °C), the supernatant was passed through a tandem C18 SPE cartridge (Waters, Milford, MA, USA) and dried under nitrogen gas for about 12 h. Subsequently, the lysate was re-suspended in 1 mL methanol and the solution filtered through a 0.45 μm organic ultrafine filter (Millipore, Shanghai, China). All the above operations were performed under minimal light. The 0.2 uL samples were utilized to run the Ultra Performance Liquid Chromatography (UPLC) (Waters).

### 4.6. GA Treatment

Exogenous GA was applied to the plants by spraying a 1:1000 aqueous dilution of 100 mM GA3 (Sigma-Aldrich, St. Louis, MO, USA) dissolved in absolute ethanol. Spraying was carried out every three days until flower bud emergence was detected.

## 5. Conclusions

**Figure 5:**
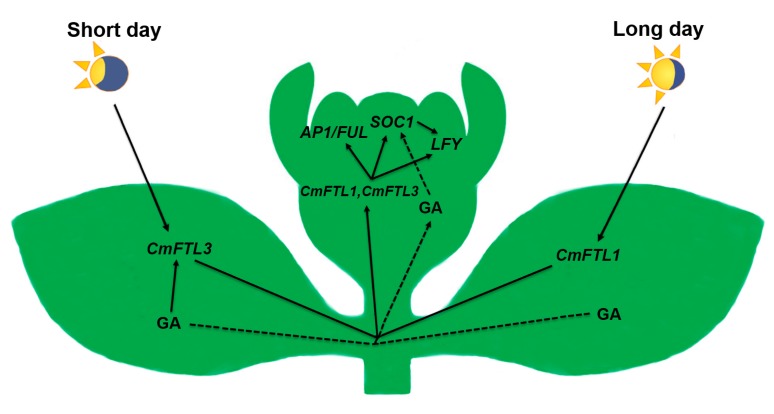
A model of the floral induction regulatory networks operating in chrysanthemum ‘Jinba’ plants grown under LD or SD.

The transcriptomic analysis and the other data of the spontaneous mutant of chrysanthemum showed that higher GA contents can affect the expression of *SOC1* under LD conditions for regulating flowering; under SD conditions, the photoperiod pathway majorly acted to determine flowering time through *CmFTL3*, and the GA signaling pathway played a subsidiary role for flowering (Figure 5).

## Figures and Tables

**Table 1:** Summary statistics of the RNA-Seq data.

Samples	Total Raw Reads	Total Clean Reads	Total Clean Nucleotides (nt)	Q20 Percentage	Q30 Percentage	N Percentage	GC Percentage
WT-SD	117,328,048	114,265,744	5,141,958,480	97.30%	91.39%	0.00	43.19%
WT-LD	117,809,964	114,693,344	5,161,200,480	97.19%	91.16%	0.00	43.59%
M-SD	121,981,564	118,657,316	5,339,579,220	97.16%	91.08%	0.00	43.18%
M-LD	120,340,980	116,781,172	5,255,152,740	97.19%	91.18%	0.00	43.53%

Total Reads and Total Nucleotides are actually clean reads and clean nucleotides; Total Nucleotides should be more than contract provision; Q20/Q30percentage is the proportion of nucleotides with a quality value larger than 20/30; N percentage is the proportion of unknown nucleotides in clean reads; GC percentage is the proportion of guanidine and cytosine nucleotides among total nucleotides. WT-LD, the ‘Jinba’ wild type-LD. WT-SD, the ‘Jinba’ wild type-SD. M-LD, the ‘Jinba’ mutant-LD. M-SD, the ‘Jinba’ mutant-SD.

**Table 2:** Differentially transcribed flowering time-related genes detected in ‘Jinba’ WT and M plants grown under LD or SD.

Gene	Annotation	WT-SD RPKM	WT-LD RPKM	M-SD RPKM	M-LD RPKM	Fold Change							
						M-SD/WT-SD	*p* Value	WT-SD/WT-LD	*p* Value	M-SD/M-LD	*p* Value	M-LD/WT-LD	*p* Value
Photoperiod pathway													
CL80.Contig1l	*CmTOC1*	2.64	1.30	3.51	1.50	1.33	4.0 × 10^−2^	2.04	1.0 × 10^−4^	2.36	2.0 × 10^−7^	1.15	5.2 × 10^−1^
CL6838.Contig1	*PRR5*	36.32	33.30	52.36	26.07	1.44	1.8 × 10^−45^	1.09	3.0 × 10^−3^	2.01	4.0 × 10^−133^	0.78	3.0 × 10^−14^
CL64.Contig1	*LHY*	3.25	2.35	4.20	1.65	1.29	2.0 × 10^−2^	1.38	1.0 × 10^−2^	2.54	2.0 × 10^−12^	0.70	2.0 × 10^−2^
CL64.Contig2	*LHY*	4.57	3.29	6.34	2.30	1.39	4.0 × 10^−4^	1.39	3.0 × 10^−3^	2.76	7.0 × 10^−20^	0.70	7.0 × 10^−2^
Unigene30909	*LHY*	3.74	1.97	5.06	2.43	1.35	3.0 × 10^−3^	1.90	7.0 × 10^−7^	2.09	8.0 × 10^−11^	1.23	1.5 × 10^−1^
CL7048.Contig2	*CONSTANS-like*	10.17	9.32	14.99	6.42	1.47	2.3 × 10^−9^	1.09	1.4 × 10^−1^	2.33	2.0 × 10^−36^	0.69	2.0 × 10^−7^
CL10258.Contig3	*CONSTANS-like*	41.44	23.72	54.60	13.61	1.38	6.8 × 10^−19^	1.68	1.0 × 10^−29^	3.74	2.0 × 10^−170^	0.62	9.0 × 10^−16^
Unigene16657	*CONSTANS-like*	93.59	77.41	100.21	43.79	1.07	5.0 × 10^−4^	1.21	4.0 × 10^−19^	2.29	1.0 × 10^−260^	0.57	3.5 × 10^−107^
GA biosynthesis and signaling													
CL10783.Contig2	*Ent*-copalyl diphosphate synthase (*CPS*)	0.09	0.79	2.06	0.28	22.89	2.0 × 10^−6^	0.11	1.5 × 10^−2^	7.36	9.5 × 10^−5^	0.35	1.3 × 10^−1^
CL2973.Contig1	Gibberellin 20-oxidase (*GA20ox*)	38.62	7.74	30.22	18.62	0.78	6.0 × 10^−14^	4.99	6.0 × 10^−261^	1.62	2.0 × 10^−34^	2.41	1.0 × 10^−54^
CL8331.Contig2	Gibberellin 2-oxidase (*GA2ox*)	0.14	3.94	0.20	0.92	1.38	5.6 × 10^−1^	0.04	2.0 × 10^−40^	0.21	8.0 × 10^−6^	0.23	7.0 × 10^−19^
Unigene27395	Gibberellin receptor (*GID1*)	3.36	3.19	2.36	8.18	0.70	1.2 × 10^−2^	1.05	7.1 × 10^−1^	0.29	5.0 × 10^−28^	2.56	1.0 × 10^−18^
Unigene27748	*GAI*	14.43	45.74	12.22	23.59	0.85	1.0 × 10^−3^	0.32	2.0 × 10^−214^	0.52	2.0 × 10^−47^	0.52	6.0 × 10^−89^
Flowering integrators													
CL1484.Contig1	*CmFTL1*	0.39	12.17	0.41	12.12	0.93	8.9 × 10^−1^	0.03	6.0 × 10^−72^	0.03	3.0 × 10^−72^	1.0	9.7 × 10^−1^
CL1484.Contig2	*CmFTL3*	16.88	1.68	31.81	2.08	1.88	6.0 × 10^−51^	3.94	5.0 × 10^−148^	15.32	0	1.23	1.69 × 10^−1^
Unigene40592	*SOC1*	8.68	3.60	17.65	16.82	2.03	2.0 × 10^−21^	2.41	9.0 × 10^−15^	1.05	4.5 × 10^−1^	4.67	5.0 × 10^−57^
Unigene25380	*FLO/LFY-like*	0.75	1.28	0.57	8.50	0.76	3.3 × 10^−1^	0.58	2.0 × 10^−2^	0.07	1.0 × 10^−71^	6.66	2.0 × 10^−50^
Unigene23898	*AP1/FUL like*	47.87	1.54	76.84	2.03	1.61	1.0 × 10^−56^	31.12	0	37.89	0	1.32	1.3 × 10^−1^
Aging, ambient temperature and autonomous pathway													
Unigene29044	*SPL5*	1.30	9.51	0.40	7.11	0.33	2.0 × 10^−3^	0.14	2.0 × 10^−33^	0.06	1.7 × 10^−33^	0.75	8.0 × 10^−3^
CL14613.Contig14	*FLC-like*	1.01	1.59	2.96	1.60	2.93	1.0 × 10^−7^	0.63	6.0 × 10^−2^	1.85	8.0 × 10^−4^	1.00	9.9 × 10^−1^
CL8773.Contig1	*FRIGIDA*	1.03	0.89	1.70	0.63	1.92	4.0 × 10^−5^	0.86	4.0 × 10^−1^	2.70	1.3 × 10^−8^	0.61	1.0 × 10^−2^
Unigene20776	*SVP*	42.08	308.77	23.50	167.50	0.56	2.0 × 10^−45^	0.14	0	0.14	0	0.54	0

Significant differences were determined with *p* < 0.001, up-regulation expression ratio >2.0 and down-regulation expression ratio <0.5. RKPM: reads per kilobase per million mapped reads. WT-SD, the ‘Jinba’ wild type under short day. WT-LD, the ‘Jinba’ wild type under long day. M-SD, the ‘Jinba’ mutant under short day. M-LD, the ‘Jinba’ mutant under long day.

## Supplementary Materials

Supplementary materials can be found at www.mdpi.com/1422-0067/18/6/1259/s1.
