# Supplementary material for: Gibberellic Acid Signaling Is Required to Induce Flowering of Chrysanthemums Grown under Both Short and Long Days
Source: Int J Mol Sci. 2017 Jun 12;18(6):1259. doi: 10.3390/ijms18061259 (PMC5486081; doi:10.3390/ijms18061259)
Supplement: Supplementary file 1 [file ijms-18-01259-s001.pdf]

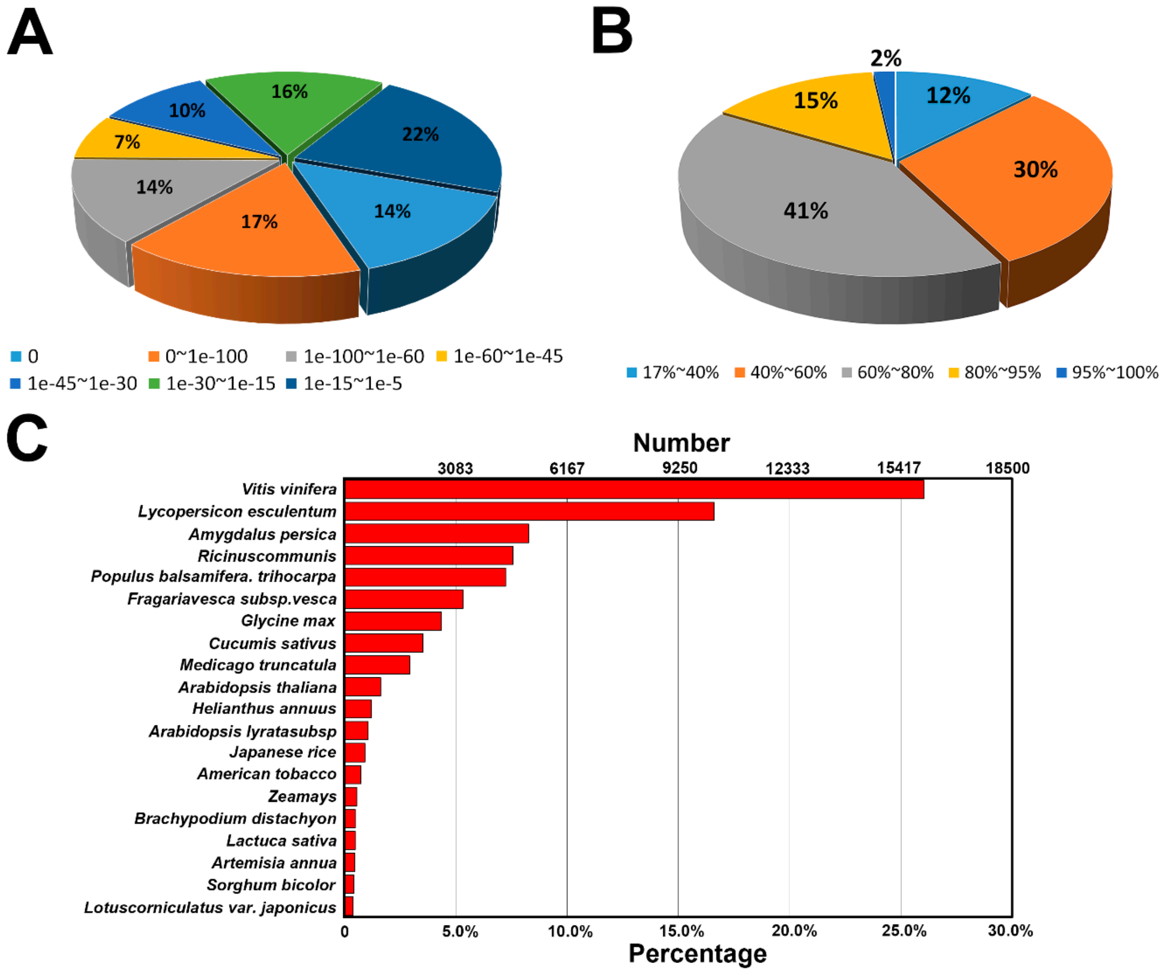

Figure S1. Chrysanthemum sequences acquired by RNA-Seq. (A) Homology of chrysanthemum unigenes to those represented in the Nr database. (B) Similarity distribution of the result against Nr database. (C) The match between differentially transcribed chrysanthemum unigenes and sequences derived from other plant species.

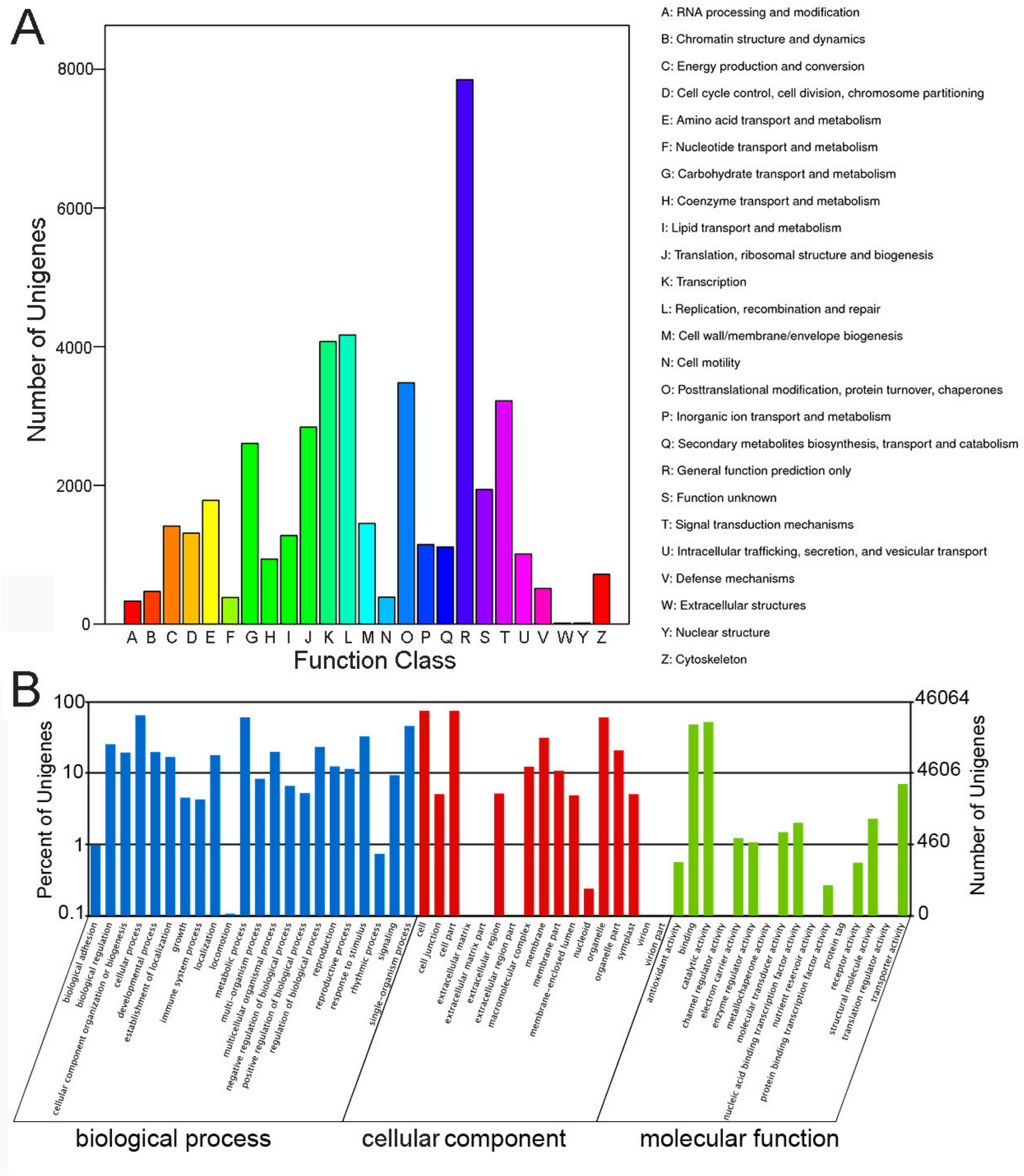

Figure S2 COG and GO classification of differentially transcribed chrysanthemum unigenes. (A) COG-based functional classification of differentially transcribed chrysanthemum unigenes. (B) GO classification of differentially transcribed chrysanthemum unigenes.

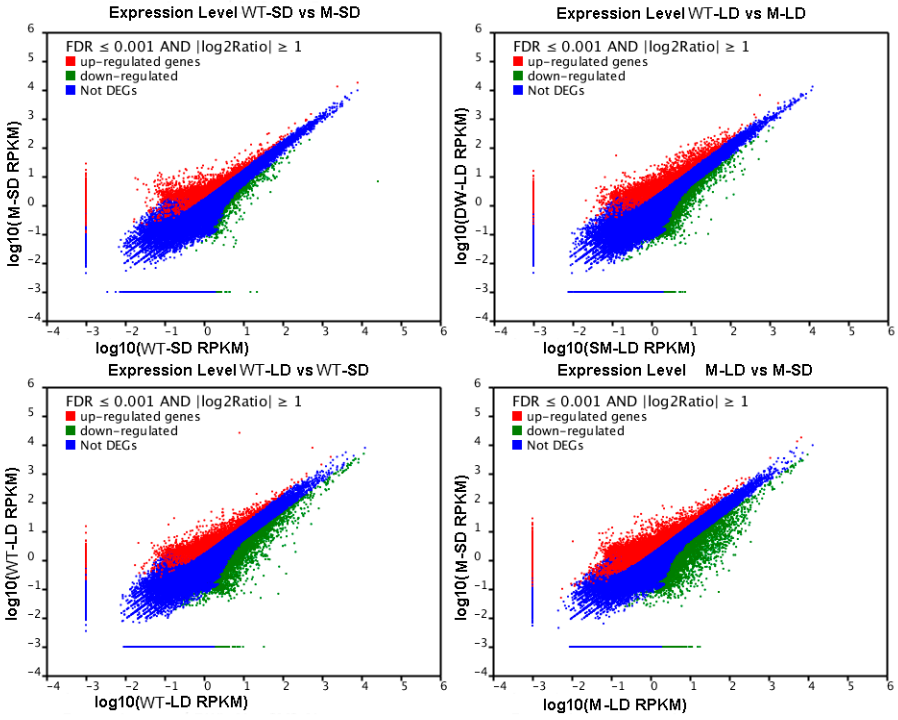

Figure S3 reads per kilobase per million mapped reads. FDR: false discovery rate. The axes indicate the frequency of recovery of each sequence. WT-SD represents wild type plants under short day conditions, WT-LD represents wild type plants under long day conditions, M-SD represents mutant plants under short day conditions, M-LD represents mutant plants under long day conditions. (Red: up-regulated, green: down-regulated, blue: not differentially transcribed).

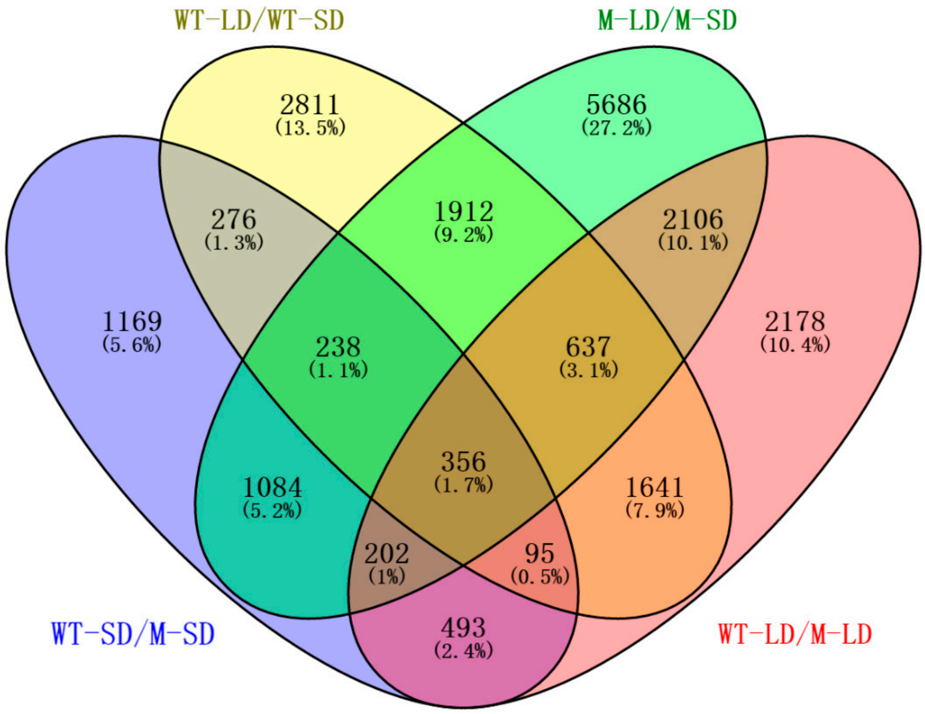

Figure S4 Venn diagram to classify differentially expressed genes in 'Jinba' WT and M plants under SD and LD.

Table S1. List of assembled transcripts up-regulated or down-regulated (> or < 2.0 fold) in WT and M chrysanthemum plant under SD and LD

	M-SD /WT-SD					WT-SD /WT-LD					M-SD/M-LD					M-LD /WT-LD					
**Gene ID**	**M-SD**	**WT-SD**	**ratio**	**p value**	**FDR**	**WT-SD**	**WT-LD**	**ratio**	**p value**	**FDR**	**M-SD**	**M-LD**	**ratio**	**p value**	**FDR**	**M-LD**	**WT-LD**	**ratio**	**p value**	**FDR**	**Annotation**
**Photoperiod pathway**																					
CL80.Contig1_All	3.51	2.64	1.33	**0.044**	0.133	2.64	1.3	2.04	1E-04	8E-04	3.51	1.5	2.36	2E-07	2E-06	1.5	1.3	**1.15**	0.521	0.66	Chrysanthemum seticuspe f.boreale CsTOC1 mRNA for timing of cab expression 1-like
CL6838.Contig1_All	52.36	36.32	1.44	2E-45	2E-43	36.32	33.3	**1.09**	0.003	0.014	52.36	26.07	2.01	#####	#####	26.07	33.3	0.78	3E-14	0	Two-component response regulator-like APRR5
CL64.Contig1_All	4.2	3.25	1.29	**0.0204**	0.074	3.25	2.35	**1.38**	0.014	0.048	4.2	1.65	2.54	2E-12	3E-11	1.65	2.35	**0.7**	0.022	0.07	Protein LHY
CL64.Contig2_All	6.34	4.57	1.39	**0.0004**	0.003	4.57	3.29	**1.39**	0.003	0.013	6.34	2.3	2.76	#####	1E-18	2.3	3.29	**0.7**	0.007	0.03	Protein LHY
Unigene30909_All	5.06	3.74	1.35	**0.0026**	0.014	3.74	1.97	1.9	7E-07	7E-06	5.06	2.43	2.09	8E-11	1E-09	2.43	1.97	**1.23**	0.154	0.29	Chrysanthemum seticuspe f. boreale CsLHY mRNA for late elongated hypocotyl-like
CL7048.Contig2_All	14.99	10.17	1.47	2E-09	5E-08	10.17	9.32	**1.09**	0.141	0.286	14.99	6.42	2.33	2E-36	7E-35	6.42	9.32	0.69	2E-07	0	Zinc finger protein CONSTANS-LIKE 9
CL10258.Contig3_All	54.6	41.44	1.38	7E-19	3E-17	41.44	23.72	1.68	1E-29	4E-28	54.6	13.61	3.74	#####	#####	13.61	23.72	0.62	9E-16	0	Zinc finger protein CONSTANS-LIKE 16
Unigene16657_All	100.21	93.59	1.07	0.0005	0.004	93.59	77.41	1.21	4E-19	9E-18	100.21	43.79	2.29	#####	#####	43.79	77.41	0.57	#####	0	Zinc finger protein CONSTANS-LIKE 16
**GA biosynthesis and signaling**																					
CL2973.Contig1_All	30.22	38.62	0.78	6E-14	2E-12	38.62	7.74	4.99	#####	#####	30.22	18.62	1.62	2E-34	7E-33	18.62	7.74	2.41	1E-54	0	gibberellin 20-oxidase2 [Chrysanthemum x morifolium]
CL8331.Contig2_All	0.2	0.14	1.38	**0.5578**	0.718	0.14	3.94	0.04	2E-40	7E-39	0.2	0.92	0.21	8E-06	5E-05	0.92	3.94	0.23	7E-19	0	Gibberellin 2-beta-dioxygenase 1

Unigene27395_All	2.36	3.36	0.7	**0.0121**	0.05	3.36	3.19	**1.05**	0.705	0.813	2.36	8.18	0.29	5E-28	1E-26	8.18	3.19	2.56	1E-18	0	Gibberellin receptor GID1
Unigene27748_All	12.22	14.43	0.85	**0.0011**	0.007	14.43	45.74	0.32	#####	#####	12.22	23.59	0.52	2E-47	1E-45	23.59	45.74	0.5	6E-89	0	DELLA protein GAI
**Flowering integrators**																					
CL1484.Contig1_All	0.41	0.39	0.93	**0.8889**	0.951	0.39	12.17	0.03	6E-72	4E-70	0.41	12.12	0.03	3E-72	2E-70	12.12	12.17	**1**	0.967	0.97	flowering locus T-Like 1 protein [Chrysanthemum x morifolium]
CL1484.Contig2_All	31.81	16.88	1.88	6E-51	7E-49	16.88	1.68	3.94	#####	#####	31.81	2.08	15.32	0	0	2.08	1.68	**1.23**	0.169	0.31	flowering locus T-Like 3 protein [Chrysanthemum x morifolium]
Unigene40592_All	17.65	8.68	2.03	2E-21	1E-19	8.68	3.6	2.41	9E-15	2E-13	17.65	16.82	**1.05**	0.455	0.576	16.82	3.6	4.67	5E-57	0	MADS-box protein SOC1
Unigene25380_All	0.57	0.75	0.76	**0.3343**	0.524	0.75	1.28	**0.58**	0.02	0.066	0.57	8.5	0.07	1E-71	8E-70	8.5	1.28	6.66	2E-50	0	Chrysanthemum x morifolium FLO/LFY-like protein gene
Unigene23898_All	76.84	47.87	1.61	1E-56	2E-54	47.87	1.54	31.12	0	0	76.84	2.03	37.89	0	0	2.03	1.54	1.32	0.126	0.25	APETALA1 and FRUITFULL like protein [Chrysanthemum seticuspe f. boreale]
**Aging, ambient temperature and autonomous pathway**																					
Unigene29044_All	0.4	1.3	0.33	**0.0024**	0.014	1.3	9.51	0.14	2E-33	7E-32	0.4	7.11	0.06	2E-33	0.03	7.11	9.51	**0.75**	0.008	0.03	Squamosa promoter-binding-like protein 5
CL14613.Contig14_All	2.96	1.01	2.93	1E-07	2E-06	1.01	1.59	**0.63**	0.056	0.144	2.96	1.6	1.85	8E-04	0.994	1.6	1.59	**1**	0.992	0.99	FLC-like
CL8773.Contig1_All	1.7	1.03	1.92	4E-05	4E-04	1.03	0.89	**0.86**	0.396	0.568	1.7	0.63	2.7	1E-08	0.044	0.63	0.89	**0.61**	0.013	0.04	Protein FRIGIDA
Unigene20776_All	23.5	42.08	0.56	2E-45	2E-43	42.08	308.8	0.14	0	0	23.5	167.5	0.14	0	0	167.5	308.8	0.54	0	0	MADS-box protein SVP

The bold repreats those genes are not differentially expressed and P>0.001.

Table S2 Transcription factor genes, arranged by family, which were differentially transcribed in WT and M chrysanthemum plants grown under SD and LD.

List of difference expression MYB family (> 2 fold or < 0.5, p<0.001) in WT-SD /WT-LD chrysanthemum plants					
unigene	WT-SD /WT-LD				Nr Annotation
	WT-SD	WT-LD	ratio	P-value	description
CL897.Contig4_All	0.5003	0.052	9.621154	8.50214E-05	MYB transcription factor [Camellia sinensis]
Unigene2122_All	21.0682	6.3949	3.294532	1.6812E-75	PREDICTED: transcription factor MYB59-like [Vitis vinifera]
Unigene39827_All	3.1695	0.5882	5.388473	3.65158E-14	PREDICTED: transcription factor MYB75-like [Vitis vinifera]
CL4324.Contig1_All	4.6377	0.3433	13.50918	8.13738E-13	PREDICTED: transcription factor MYB59-like [Vitis vinifera]
CL10138.Contig2_All	1.2649	0.1315	9.619011	8.50214E-05	PREDICTED: transcription factor MYB59-like [Vitis vinifera]
CL9908.Contig3_All	1.3899	0.6442	2.15756	7.39666E-06	MYB-like DNA-binding protein [Catharanthus roseus]
Unigene1626_All	16.8572	4.9234	3.423894	7.07306E-15	PREDICTED: transcription factor MYB28-like, partial [Solanum lycopersicum]
Unigene12681_All	21.0682	6.3949	3.294532	1.6812E-75	PREDICTED: transcription factor MYB59-like [Vitis vinifera]
CL5312.Contig2_All	2.6261	0	0	9.28438E-09	MYB2 [Chrysanthemum x morifolium]
Unigene45180_All	1.8665	0	0	1.62678E-06	MYB-related protein [Lotus japonicus]
Unigene38746_All	4.8071	0.3463	0.072039	4.1544E-21	MYB2 [Chrysanthemum x morifolium]
Unigene17629_All	2.3789	0.2179	0.091597	2.78432E-05	PREDICTED: myb-related protein Zm38-like [Cucumis sativus]
Unigene43294_All	4.9717	0.4758	0.095702	7.3961E-15	PREDICTED: myb-related protein 306 isoform 1 [Vitis vinifera]
CL7700.Contig2_All	2.74	0.3861	0.140912	1.18567E-05	MYB1 protein [Gerbera hybrid cultivar]
Unigene29197_All	2.4215	0.3697	0.152674	3.96808E-07	PREDICTED: transcription repressor MYB4-like [Solanum lycopersicum]
Unigene8384_All	2.9663	0.5055	0.170414	7.78016E-08	PREDICTED: myb family transcription factor APL-like [Solanum lycopersicum]
Unigene9085_All	8.862	2.3836	0.268969	1.3034E-27	MYB9A protein [Gerbera hybrid cultivar]
CL10078.Contig2_All	4.35	1.4942	0.343494	3.824E-10	MYB9A protein [Gerbera hybrid cultivar]
Unigene19056_All	10.4977	4.1772	0.397916	3.57652E-24	transcription factor DcMYB2 [Daucus carota]
CL10078.Contig1_All	6.0313	2.6342	0.436755	2.16304E-09	MYB9A protein [Gerbera hybrid cultivar]
Unigene7748_All	11.5011	5.1934	0.451557	1.61667E-22	MYB6 [Malus x domestica]
Unigene19649_All	24.9635	11.3513	0.454716	6.67332E-49	MYB2 [Chrysanthemum x morifolium]
Unigene24264_All	2.2834	1.0593	0.463913	0.000059543	Myb-like protein P [Saussurea medusa]

Unigene750_All	3.276	1.6367	0.499603	3.5217E-06	PREDICTED: myb-related protein 3R-1-like [Glycine max]

List of difference expression MYB family (> 2 fold or < 0.5, p<0.001) in M-SD /M-LD chrysanthemum plants					
unigene	M-SD /M-LD				Nr Annotation
	M-SD	M-LD	ratio	P-value	description
Unigene29981_All	3.5745	0.131	27.28626	1.46E-14	predicted protein [Populus trichocarpa]
CL10138.Contig1_All	1.4806	0.071	20.85352	0.00000619	PREDICTED: myb-related protein 305-like [Fragaria vesca subsp. vesca]
CL4324.Contig2_All	2.3897	0.2189	10.91686	0.000021	PREDICTED: transcription factor MYB48-like [Glycine max]
Unigene12681_All	27.7388	3.0731	9.026325	1.87E-206	PREDICTED: transcription factor MYB28-like, partial [Solanum lycopersicum]
Unigene39827_All	6.4769	0.7288	8.887075	2.65E-36	PREDICTED: transcription factor MYB75-like [Vitis vinifera]
CL4324.Contig1_All	6.7679	0.9037	7.4891	5.95E-15	PREDICTED: transcription factor MYB48-like [Glycine max]
CL469.Contig7_All	4.3004	0.7109	6.049233	5.63E-20	PREDICTED: transcription factor MYB59-like [Vitis vinifera]
CL10138.Contig2_All	1.8744	0.3777	4.962669	0.0000454	PREDICTED: myb-related protein 305-like [Fragaria vesca subsp. vesca]
Unigene30298_All	1.624	0.3506	4.632059	4.44E-08	PREDICTED: transcription factor MYB59-like [Vitis vinifera]
CL3505.Contig2_All	4.3229	0.9679	4.466267	0.0000172	R2R3-MYB transcription factor MYB6 [Epimedium sagittatum]
CL469.Contig4_All	2.9517	0.6848	4.31031	1.33E-18	PREDICTED: transcription factor MYB59-like [Cucumis sativus]
CL7523.Contig3_All	3.6345	0.9411	3.86197	1.06E-19	PREDICTED: myb-related protein 306 [Vitis vinifera]
CL897.Contig3_All	1.5011	0.3936	3.81377	9.51E-09	MYB transcription factor [Camellia sinensis]
Unigene7743_All	34.0525	9.2122	3.696457	8.64E-139	MYB1 [Chrysanthemum x morifolium]
CL897.Contig4_All	1.5074	0.498	3.026908	0.00000436	MYB transcription factor [Catharanthus roseus]
CL469.Contig5_All	1.9682	0.7082	2.779158	0.000000353	PREDICTED: transcription factor MYB59-like [Vitis vinifera]
Unigene2122_All	25.4937	9.3556	2.724967	4.14E-63	PREDICTED: transcription factor MYB59-like [Vitis vinifera]
CL4900.Contig3_All	0.7513	0.2951	2.545917	0.0000689	MYB-like DNA-binding protein [Catharanthus roseus]
Unigene33281_All	12.541	5.0852	2.466176	0.00000468	PREDICTED: transcription factor MYB48-like [Solanum lycopersicum]
Unigene19503_All	47.2359	19.7287	2.394273	2.34E-89	ATMYB6 [Arabidopsis lyrata subsp. lyrata]
Unigene31255_All	4.412	1.9629	2.247695	7.62E-09	PREDICTED: transcription factor MYB39 [Vitis vinifera]
CL9206.Contig1_All	3.0403	1.3615	2.233052	0.0000915	PREDICTED: transcription factor MYB46-like [Vitis vinifera]
Unigene35243_All	3.988	1.8082	2.205508	0.000000574	putative MYB DNA-binding domain superfamily protein [Zea mays]
CL9605.Contig1_All	0	0.4157	0	0.00000355	PREDICTED: transcription factor MYB1R1 [Vitis vinifera]

Unigene24264_All	0.1691	19.7025	0.008583	2.3E-198	Myb-like protein P [Saussurea medusa]
Unigene7748_All	2.5277	81.4719	0.031025	0	MYB6 [Malus x domestica]
Unigene38746_All	0.2053	3.1542	0.065088	1.67E-14	MYB2 [Chrysanthemum x morifolium]
Unigene2782_All	0.7262	8.414	0.086309	4.95E-15	MYB2 [Chrysanthemum x morifolium]
Unigene11644_All	0.644	6.6186	0.097302	1.85E-30	PREDICTED: transcriptional activator Myb-like [Solanum lycopersicum]
Unigene15592_All	2.9665	29.0025	0.102284	3.38E-165	PREDICTED: myb-related protein Myb4 [Vitis vinifera]
Unigene43294_All	0.141	1.3497	0.104468	0.000113082	PREDICTED: myb-related protein 306 isoform 1 [Vitis vinifera]
CL5312.Contig2_All	0.1996	1.8101	0.11027	0.000207348	MYB2 [Chrysanthemum x morifolium]
Unigene2194_All	17.194	133.9839	0.128329	0	MYB2 [Chrysanthemum x morifolium]
Unigene38745_All	1.002	6.017	0.166528	3.95E-23	PREDICTED: transcription factor MYB44-like [Solanum lycopersicum]
Unigene9085_All	0.2673	1.3803	0.193654	0.00000102	MYB9A protein [Gerbera hybrid cultivar]
CL10078.Contig2_All	0.2952	1.227	0.240587	0.0000622	MYB9A protein [Gerbera hybrid cultivar]
Unigene19649_All	13.995	46.5327	0.300756	1.05E-167	MYB2 [Chrysanthemum x morifolium]
Unigene8384_All	1.2454	3.7643	0.330845	0.00000841	PREDICTED: myb family transcription factor APL-like [Solanum lycopersicum]
Unigene40440_All	11.4125	31.5359	0.361889	1.68E-89	PREDICTED: myb-related protein 306 isoform 1 [Vitis vinifera]

List of difference expression MYB family (> 2 foldor < 0.5, p<0.001) in M-SD /WT-SD chrysanthemum plants					
unigene	M-SD /WT-SD				Nr Annotation
	M-SD	WT-SD	ratio	P-value	description
CL897.Contig3_All	1.5011	0.2379	6.309794	1.47406E-12	MYB transcription factor [Camellia sinensis]
CL897.Contig4_All	1.5074	0.5003	3.012992	3.4341E-06	MYB transcription factor [Catharanthus roseus]
Unigene2122_All	25.4937	9.9046	2.573925	2.38986E-59	PREDICTED: transcription factor MYB59-like [Vitis vinifera]
CL7523.Contig3_All	3.6345	1.6833	2.159152	1.38376E-09	PREDICTED: myb-related protein 306 [Vitis vinifera]
Unigene39827_All	6.4769	3.1695	2.043508	2.1909E-09	PREDICTED: transcription factor MYB75-like [Vitis vinifera]
Unigene9085_All	0.2673	2.3836	0.112141	3.07702E-14	MYB9A protein [Gerbera hybrid cultivar]
CL10078.Contig1_All	0.3416	2.6342	0.129679	4.4103E-13	MYB9A protein [Gerbera hybrid cultivar]
Unigene24264_All	0.1691	1.0593	0.159634	5.77608E-07	Myb-like protein P [Saussurea medusa]
CL10078.Contig2_All	0.2952	1.4942	0.197564	1.3032E-06	MYB9A protein [Gerbera hybrid cultivar]

Unigene35243_All	3.988	11.8858	0.335526	4.61594E-29	putative MYB DNA-binding domain superfamily protein [Zea mays]
Unigene7748_All	2.5277	5.1934	0.486714	1.05037E-09	MYB6 [Malus x domestica]

List of difference expression MYB family (> 2 foldor < 0.5, p<0.001) in M-LD /WT-LD chrysanthemum plants					
unigene	M-LD /WT-LD				Nr Annotation
	M-LD	WT-LD	ratio	P-value	description
CL897.Contig4_All	0.052	0.498	9.576923	9.97866E-05	MYB transcription factor [Catharanthus roseus]
Unigene24264_All	2.2834	19.7025	8.62858	1.6277E-119	Myb-like protein P [Saussurea medusa]
Unigene7748_All	11.5011	81.4719	7.083835	0	MYB6 [Malus x domestica]
Unigene38745_All	0.928	6.017	6.483836	2.04446E-23	PREDICTED: transcription factor MYB44-like [Solanum lycopersicum]
Unigene2194_All	28.0579	133.9839	4.775265	0	MYB2 [Chrysanthemum x morifolium]
Unigene2782_All	2.1654	8.414	3.885656	2.63222E-08	MYB2 [Chrysanthemum x morifolium]
Unigene15592_All	8.1656	29.0025	3.55179	9.02516E-82	PREDICTED: myb-related protein Myb4 [Vitis vinifera]
Unigene23071_All	9.2704	31.24	3.369865	1.4442E-271	MYB2 [Chrysanthemum x morifolium]
CL12917.Contig1_All	0.4277	1.3161	3.077157	8.51066E-05	MYB transcription factor [Camellia sinensis]
Unigene11644_All	2.4853	6.6186	2.663099	4.0535E-11	PREDICTED: transcriptional activator Myb-like [Solanum lycopersicum]
Unigene2122_All	3.8165	9.3556	2.451356	3.9992E-20	PREDICTED: transcription factor MYB59-like [Vitis vinifera]
Unigene28715_All	1.2278	2.6049	2.1216	0.000088745	PREDICTED: transcription factor MYB59-like [Vitis vinifera]
Unigene9085_All	8.862	1.3803	0.155755	1.73787E-40	MYB9A protein [Gerbera hybrid cultivar]
CL10078.Contig1_All	6.0313	1.1472	0.190208	5.11118E-22	MYB9A protein [Gerbera hybrid cultivar]
Unigene43294_All	4.9717	1.3497	0.271477	4.09524E-08	PREDICTED: myb-related protein 306 isoform 1 [Vitis vinifera]
CL10078.Contig2_All	4.35	1.227	0.282069	3.15932E-12	MYB9A protein [Gerbera hybrid cultivar]
CL14329.Contig5_All	2.4976	0.8093	0.324031	1.06007E-06	PREDICTED: transcription factor MYB46-like [Vitis vinifera]
CL7523.Contig3_All	2.7567	0.9411	0.341386	2.66922E-11	PREDICTED: myb-related protein 306 [Vitis vinifera]
CL469.Contig7_All	2.0155	0.7109	0.352716	1.61317E-05	PREDICTED: transcription factor MYB59-like [Vitis vinifera]
CL9206.Contig1_All	3.422	1.3615	0.397867	4.7193E-06	PREDICTED: transcription factor MYB46-like [Vitis vinifera]
Unigene19056_All	10.4977	4.613	0.43943	5.1622E-20	transcription factor DcMYB2 [Daucus carota]
Unigene12681_All	6.3949	3.0731	0.480555	4.00436E-12	PREDICTED: transcription factor MYB28-like, partial [Solanum lycopersicum]

Table S3. qRT-PCR primer combinations used for quantifying the transcription of WT and M plants.

Gene	Annotation	Squence
CL7048.Contig2_All	*COL*	ATCCGCTCTTGCCTTTCGT
		TTCGTCTATGCTTCTAATGGGTG
CL10258.Contig3_All	*COL*	GGCGGTGAGGGTTTGTCTA
		GTGCTCGGTGGTATTGTGCT
Unigene16657_All	*COL*	TGTTTGTTTCTGGAACGAGGG
		GTGGTATTGTGCCGCTGATG
Unigene40592_All	*SOC1*	CCATGTCCAGAAGATGTCCA
		AGCCAACTGCCTCATATGCT
CL1484.Contig1_All	*FTL1*	AATCGTGTGCTATGAGAGCC
		GCTTGTAACGTCCTCTTCATGC
CL1484.Contig2_All	*FTL3*	CTATGAGAGCCCAAGGCCATCAATG
		TGATGTTCGTGCTTTCAATATGTAT
Unigene23898_All	*AP1/FUL*	CGGGTGACGTTGAAAAGAAT
		GCATGTTCCAAAGTCCACCT
Unigene25380_All	*LFY*	AGGCCTTTGCAAGAGGAAAT
		TGAAAGCAGCAATGGATCAG
CL2973.Contig1_All	*GA20ox*	TTGCTTACATCGAGCTGTGG
		CATATCGGCTCGGTAATGCT
CL8331.Contig2_All	*GA2ox*	AACCATGGAGTCCCATTTGA
		CTGAGCCAAGGGCAATAGAG
Unigene27395_All	*GID1*	ATGGGCTTGACTTTGTTC
		CTCGGTCATTACACCTTTCAG
Unigene27748_All	*GAI*	CCAATGCCTGTCAGCCTTAA
		CCGCAAAATGGTGTAGTTCAA
